# Association of wall shear stress with intracranial aneurysm rupture: systematic review and meta-analysis

**DOI:** 10.1038/s41598-017-05886-w

**Published:** 2017-07-13

**Authors:** Geng Zhou, Yueqi Zhu, Yanling Yin, Ming Su, Minghua Li

**Affiliations:** 1 Department of Diagnostic and Interventional Radiology, Shanghai Jiao Tong University Affiliated Sixth People’s Hospital, Shanghai, China; 2 Department of Anesthesiology, The Military General Hospital of Beijing PLA, Beijing, China; 3 Shandong Academy of Chinese Medicine, 7 West Yan Zi Shan Road, Lixia, Jinan China

## Abstract

To evaluate the relationship between wall shear stress (WSS) magnitude and cerebral aneurysm rupture and provide new insight into the disparate computational fluid dynamics (CFD) findings concerning the role of WSS in intracranial aneurysm (IA) rupture. A systematic electronic database (PubMed, Medline, Springer, and EBSCO) search was conducted for all accessible published articles up to July 1, 2016, with no restriction on the publication year. Abstracts, full-text manuscripts, and the reference lists of retrieved articles were analyzed. Random effects meta-analysis was used to pool the complication rates across studies. Twenty-two studies containing CFD data on 1257 patients with aneurysms were included in the analysis. A significantly higher rate of low WSS (0–1.5 Pa) was found in ruptured aneurysms (odds ratio [OR] 2.17; 95% confidence interval [CI], 1.73–2.62). The pooled analyses across 14 studies with low WSS showed significantly lower mean WSS (0.64 vs. 1.4 Pa) (p = 0.037) in the ruptured group. This meta-analysis provides evidence that decreased local WSS may be an important predictive parameter of IA rupture.

## Introduction

With recent improvements and more diffuse use of cerebrovascular imaging methods, the detection of unruptured cerebral aneurysms has increased, and the incidence is thought to be as high as 7%^1,2^.

Whether to intervene or conservatively follow an incidentally found unruptured aneurysm (UA) remains a decisional challenge for clinicians and involves assessment of the rupture risk for each individual aneurysm in comparison with the morbidity risk carried by current treatments.

Accordingly, a good estimation of the probability of rupture of an intracranial aneurysm (IA) is of high value. Hemodynamics plays a central role throughout IA natural history, and shear stress has emerged as an important determinant of arterial physiological characteristics^3^.

Over four decades, much interest has been centered on the parameter of wall shear stress (WSS) from computational fluid dynamics (CFD) analysis. However, these extensive studies reported divergent and controversial findings on the correlation of low and high WSS with rupture^4^.

To facilitate a more accurate predictive assessment for IA rupture risk by CFD, we explored the relationship between WSS magnitude and cerebral aneurysm rupture using data obtained from the current literature, and provide new insight into the disparate CFD findings on the role of WSS in IA rupture.

## Methods

The medical literature on hemodynamic analysis for IAs was reviewed up to July 1, 2016. Title, abstract, key words, and free text were searched using combinations of the following terms: “intracranial” or “cerebral,” “aneurysm” and “hemodynamics” or “computational fluid dynamic” and “wall shear stress” in PubMed, Medline, Springer, and EBSCO.

In addition, references from the publications obtained were checked for additional studies. Two investigators (G.Z. and M.S.) performed the systematic literature search.

### Inclusion and Exclusion Criteria

The inclusion criteria were as follows: 1) presence of data on WSS by CFD simulation, 2) English language study, and 3) at least five patients per study. The exclusion criteria were as follows: 1) study not published in full, and 2) editorials, letters, review articles, guidelines, case reports, *in vitro* or cadaveric studies, and studies on animal experimentation.

### Data Extraction

Two authors (G.Z. and M.S.) extracted the data independently, including study characteristics (name of the first author, year of publication, parameters, and aneurysmal characteristic including aneurysm status and morphology). The unit for WSS was converted and unified into Pascal (Pa) in our analysis.

Based on previous studies, we set two WSS threshold ranges in our analysis of 0–1.5 and 0–2.0 Pa as WSS ranges to cause pathological changes and to define pathological low WSS. This threshold value is considered responsible for endothelial cell dysfunction causing arterial wall remodeling^5^.

We studied the effect of aneurysm size on WSS value, stratifying aneurysms as small (<10 mm) and large (≥10 mm). Results were graphically represented by using scatter plots with regression. To further investigate hemodynamic risk factors contribute to aneurysm rupture, aneurysms were classified according to asymptomatic/symptomatic and regular/irregular (blebs, aneurysm wall protrusions, or multiple lobes were present). We performed an analysis for publications that adopted patient-specific inflow boundary conditions as patient-specific measurements may be necessary for accurate and reliable calculation of WSS.

### Quality Assessment and Statistical Analysis

Two authors (G.Z. and M.S.) separately graded the quality of the studies using the STROBE (Strengthening the reporting of observational studies in epidemiology) checklist (22 items). Disagreements were resolved through discussion. Meta-analysis was performed using the STATA 13 statistical package (StataCorp, College Station, Texas, USA). We pooled data for random effects meta-analysis and calculated weighted mean differences and the 95% confidence intervals (CI). Dichotomous variables are presented as odds ratios (ORs) with 95% CI. Results are presented as the mean ± SD. Matched analysis was performed as appropriate. Significance was set at p < 0.05. Heterogeneity across studies was evaluated using the I^2^ statistic. We assessed publication bias using funnel plots and by applying the Egger test.

## Results

PubMed, Medline, Springer, and EBSCO database searches using the abovementioned key words yielded 393, 240, 466, and 131 results, respectively. The results were limited to journal articles; duplicates were removed.

After abstract screening, a total 175 full-text manuscripts were retrieved, of which 22 met the inclusion criteria^6–27^. In total, 1257 aneurysms from these 22 studies that performed CFD in search of correlations between aneurysmal hemodynamics and rupture were included in the analysis (Table 1).

**Table 1:** Characteristics of studies included in the meta-analysis.

Author, year	Ruptured/unruptured aneurysms (no.)	Ruptured/unruptured diameter (mm)	Site	Ruptured mean WSS (Pa)	Unruptured mean WSS (Pa)	P-value	Ruptured mean OSI	Unruptured mean OSI	P-value	Fluid model	Database
Cebral^14^, 2015	9/0	NA	IA	>2	NA					Newtonian	DSA
Duan^15^, 2016	6/24	7.8 ± 2.2/4.7 ± 2.1	PcomA	0.433 ± 0.242	0.901 ± 0.322	0.005	0.019 ± 0.040	0.017 ± 0.026	0.897	Newtonian	DSA
Fan^16^, 2015	16/16	7.14 ± 3.53/4.76 ± 2.19	Mirror IA	7.43 ± 4.03	11.29 ± 5.41	0.029	0.0219 ± 0.0189	0.0219 ± 0.0189	0.319	Newtonian	—
Fukazawa^17^, 2015	12/0	7.8	MCA rupture point	0.29	2.27	<0.01				Newtonian	CTA
Goubergrits^18^, 2012	7/15	3.17 ± 1.24	MCA	1.89 ± 0.81	2.1 ± 0.49	<0.001				non-Newtonian	DSA
Jing^19^, 2015	69/86	5.55/2.84	IA	0.53 ± 0.223	0.83 ± 0.284	< 0.001	0.0109 ± 0.024	0.0058 ± 0.010	<0.001	Newtonian	DSA
Jou^7^, 2008	8/18	11.0 ± 6.9/6.9 ± 3.3	IA	1.9 ± 1.1	2.6 ± 1.9	0.50				NA	DSA
Kawaguchi^20^, 2012	13/139	5.9/4.7	IA with bleb	0.49 ± 0.12	0.64 ± 0.15	< 0.01	0.38 ± 0.070	0.34 ± 0.17	NS	NA	MRA
Liu^21^, 2014	26/84	4.41 ± 2.68/5.63 ± 3.4	Par	0.7518 ± 0.36	0.7391 ± 0.29	0.855	0.0128 ± 0.013	0.0152 ± 0.016	0.947	Newtonian	DSA
Liu^22^, 2016	3/8	16.47 ± 5.28/12.04 ± 2.18	ICA	0.30 ± 0.06	0.48 ± 0.13	0.048	0.05 ± 0.04	0.04 ± 0.03	1.000	Newtonian	DSA
Lu^23^, 2011	9/9	NA	Mirror IA	6.49 ± 3.48	9.80 ± 4.12	0.015	0.0879 ± 0.0764	0.0183 ± 0.0191	0.008	Newtonian	DSA
Lv^24^, 2016	33/21	5.10/3.48	PcomA	0.52	0.80	0.001	0.02	0.01	0.05	NA	DSA
Miura^25^, 2013	43/63	5.30/5.36	MCA	7.19	9.55	0.00010	0.0165	0.0125	0.00891	Newtonian	DSA
Omodaka^26^, 2012	6/0	NA	MCA rupture point	1.10	4.96	0.031	0.0148	0.0059	0.156	Newtonian	DSA
Russell^8^, 2013	27	NA	IA with bleb	1.68 ± 1.17	2.90 ± 1.02	<0.001				Newtonian	DSA
Schneiders^27^, 2015	55/62	7.35/6.64	IA	1.14	1.70	NS	0.02	0.01	NS	NA	MRA
Shojima^49^, 2004	3/17	3.36/4.31	MCA	2.92	1.48	0.05				Newtonian	CTA
Xiang^6^, 2011	38/81	5.15 ± 2.72/4.01 ± 2.00	IA	0.33 ± 0.28	0.68 ± 0.40	<0.0001	0.016 ± 0.031	0.0035 ± 0.0044	<0.0001	Newtonian	DSA
Xu^50^, 2013	8/8	5.20 ± 1.41/4.40 ± 2.72	Mirror PcomA	0.52 ± 0.20	0.81 ± 0.21	0.012	0.0329 ± 0.0318	0.0456 ± 0.0676	0.674	Newtonian	DSA
Yu^51^, 2013	9/9	5.35/5.73	PcomA	8.11	5.27	0.024				Newtonian	DSA
Zhang^52^, 2014	20/20	4.68/3.20	ICA pair	0.28 ± 0.289	1.22 ± 1.658	0.020	0.0104 ± 0.02054	0.0061 ± 0.00871	0.156	Newtonian	DSA
Zhang^53^, 2016	108/65	5.33/4.36	PcomA	0.55 ± 0.23	0.69 ± 0.25	<0.001	0.0117	0.0082	0.038	Newtonian	DSA

Note: WSS, wall shear stress; OSI, oscillatory shear index; WSSG, WSS gradient; IA, intracranial aneurysm; ICA, internal carotid artery; Par, paraclinoid; PComA, posterior communicating artery; MCA, middle cerebral artery; NA, not available; DSA, digital subtraction angiography; CTA, computed tomographic angiography; MRA, magnetic resonance angiography; NS, not significant.

**Figure 1:**
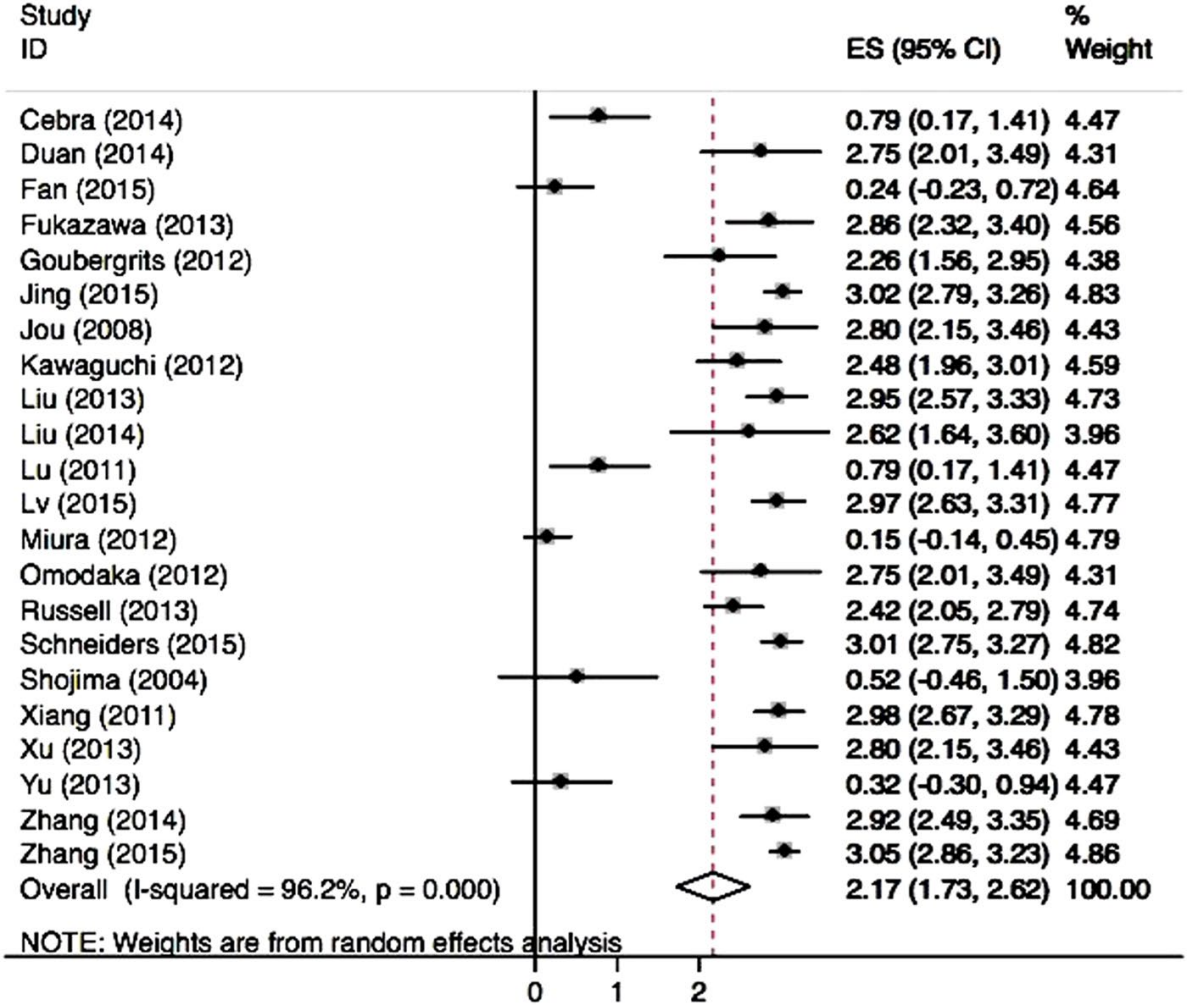
Meta-analysis of the reported low WSS rate of ruptured aneurysms.

**Figure 2:**
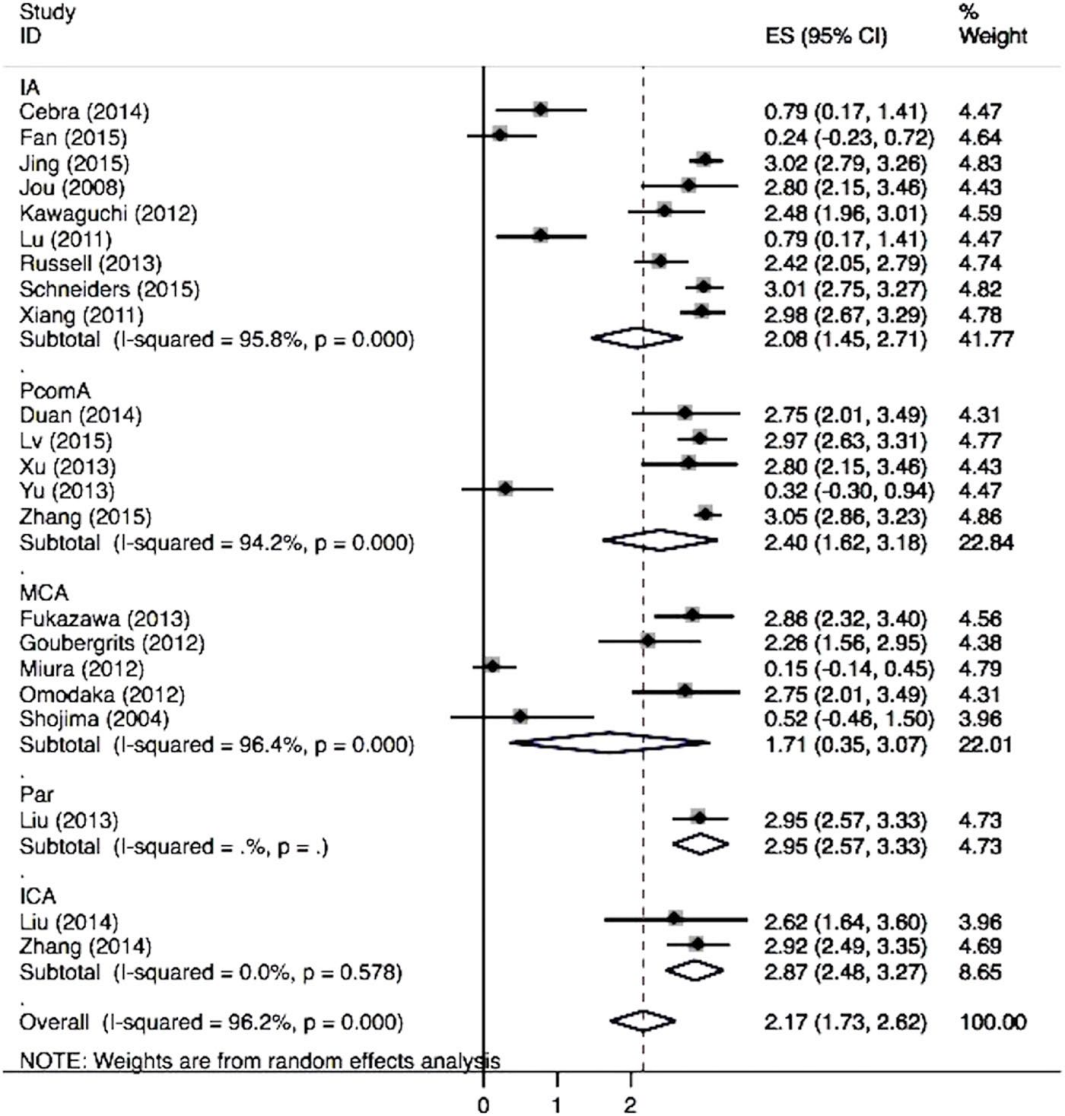
Forest plot of low WSS rate stratified by the location of aneurysm.

**Figure 3:**
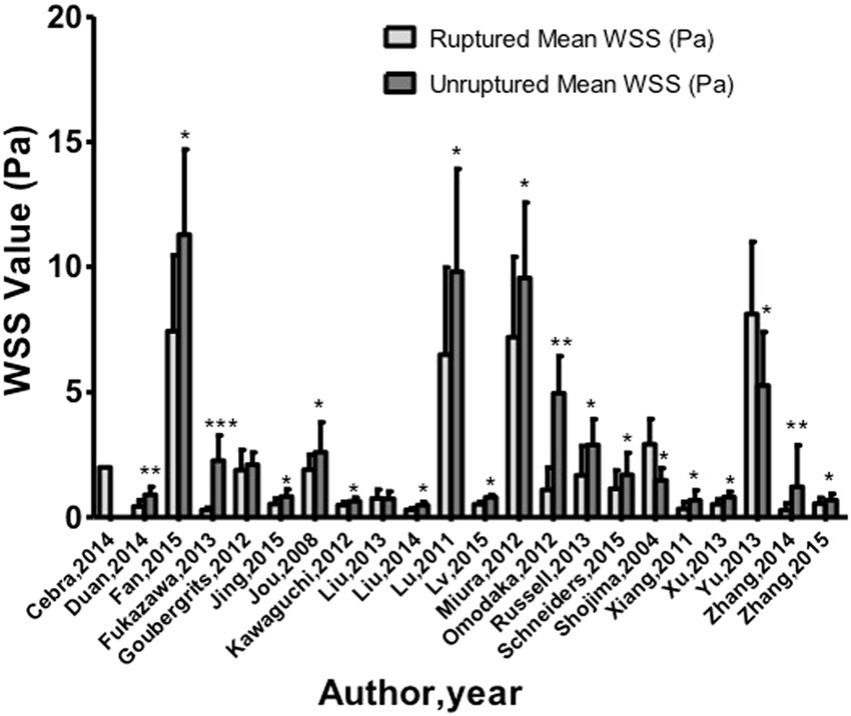
The magnitude of repoted wall shear stress (Pascal [PA]).

Our analysis showed a significantly higher rate of low WSS for ruptured aneurysms (OR 2.17; 95% CI, 1.73–2.62) (Fig. 1). Subgroup analysis found that patients with internal carotid artery aneurysms, especially posterior communicating artery (PcomA) aneurysms, had higher low WSS occurrence in ruptured cases (Fig. 2). Similarly, significantly higher rates of WSS between 0–2.0 Pa was found in ruptured aneurysms (OR 2.12; 95% CI, 0.89–5.06). Eighteen of the 22 studies demonstrated a positive correlation between low WSS and IA rupture (Fig. 3). The pooled analyses across 14 studies with low WSS (0–1.5 Pa) showed significantly lower mean WSS (0.64 vs. 1.4 Pa) (p = 0.037) in the ruptured group. PcomA aneurysms also had lower average WSS (0.51 Pa), which was lower than that of middle cerebral artery (MCA) aneurysms (1.1 Pa). We compared the WSS values based on generalized versus patient-specific profiles, the former showed significantly higher mean WSS in ruptured aneurysms (2.9 ± 3.0 Pa versus 0.67 ± 0.4 Pa, p = 0.02). For unruptured aneurysms, it was 3.68 ± 3.8 Pa versus 1.59 ± 1.7 Pa (p = 0.054).

**Figure 4:**
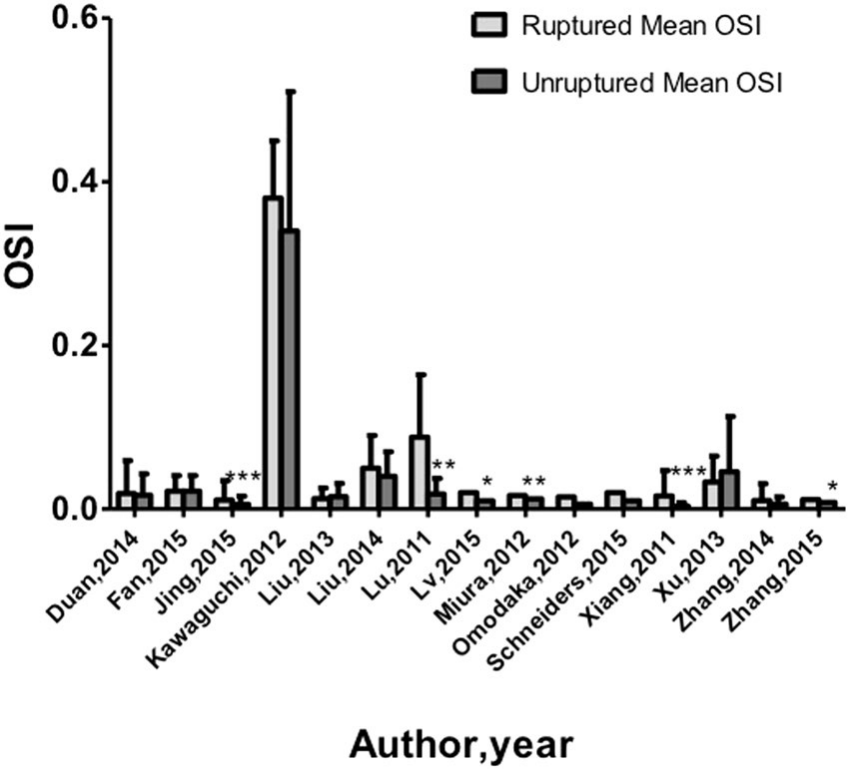
The magnitude of repoted oscillatory shear index.

**Figure 5:**
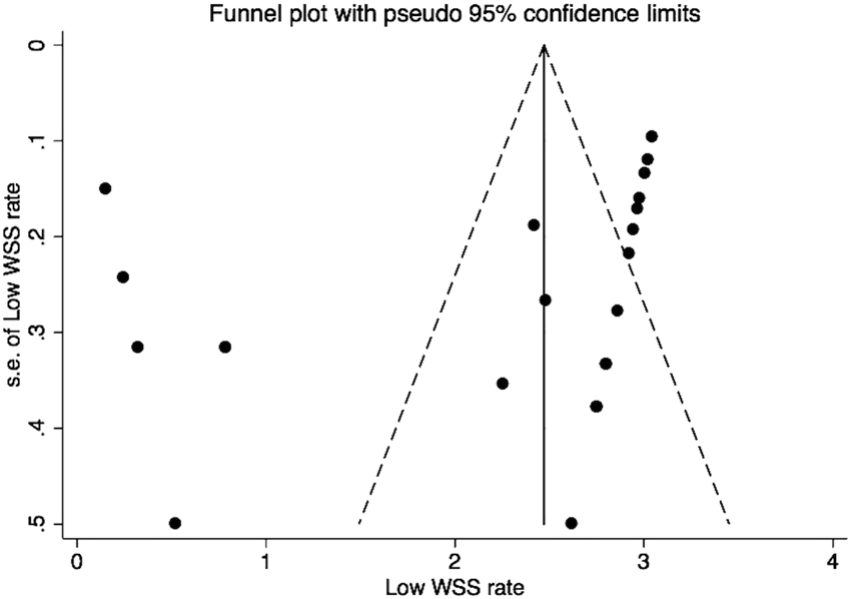
Funnel plot of the reported rate of low WSS.

**Figure 6:**
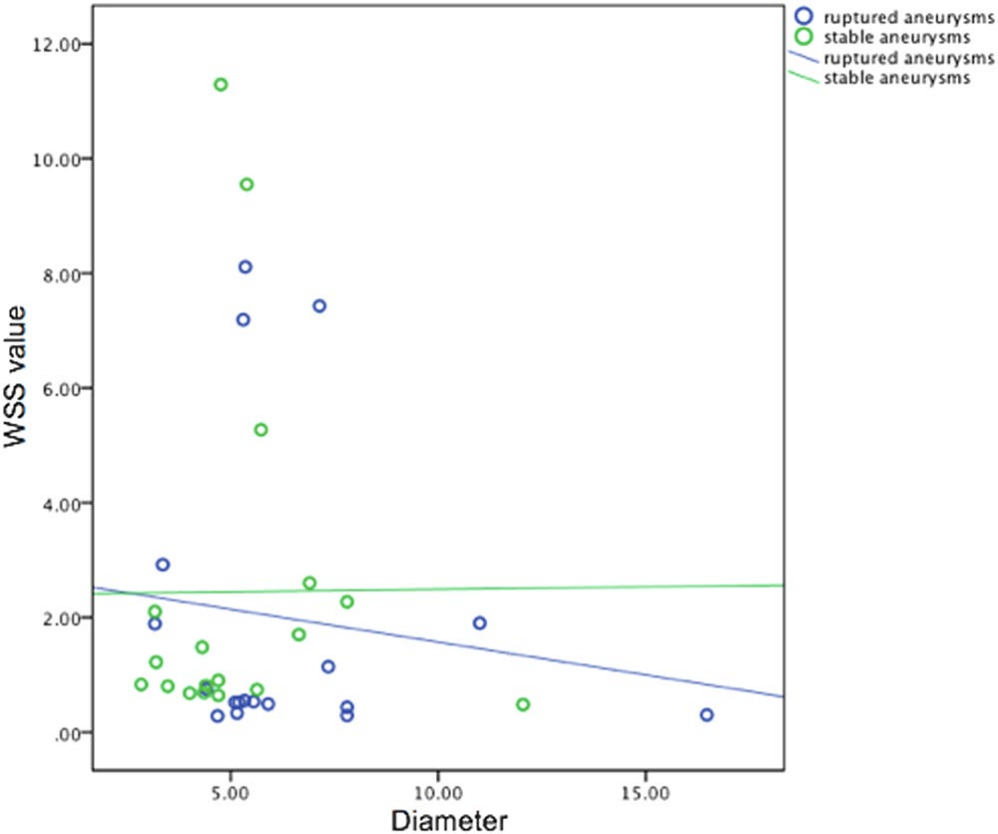
Scatterplots showing the interaction between WSS and aneurysm diameter in unruptured and ruptured cases.

Positive correlation with high oscillatory shear index (OSI) and IA rupture was found in six of 15 reports (Fig. 4). The 15 studies, involving 453 ruptured and 686 unruptured cases, were analyzed to determine the association of OSI with ruptured IA, and the pooled standardized mean difference (SMD) between the ruptured and unruptured groups showed high OSI in the ruptured group, with no significance. There was significant asymmetry in the funnel plot for articles reporting mean WSS (normalized and not normalized), and the Egger test revealed the presence of publication bias (Fig. 5). Our meta-analysis suggests that mean WSS from large ruptured aneurysm was 1.1 ± 1.1 Pa, which was insignificantly lower than patients with small ruptured aneurysms (2.1 ± 2.8 Pa) (p = 0.42). Mean WSS was 0.48 Pa for large unruptured aneurysms and 2.6 ± 3.2 Pa for their small counterparts (p = 0.53). Yet, there was a possible trend towards statistical significance (R^2^ = 0.018, p = 0.094) regarding the correlation between aneurysm and diameter in ruptured cases (Fig. 6). Our findings found irregular shape aneurysms have been shown to be characterized by lower WSS (0.86 ± 0.61 versus 1.16 ± 0.67, p = 0.33). We also demonstrated a lower WSS in the symptomatic group than in asymptomatic group (0.51 ± 0.06 versus 0.75 ± 0.08, p = 0.078). At peak systole, increase in pressure were observed in the ruptured aneurysms than unruptured ones (497 ± 164.2 Pa in ruptured aneurysms vs. 382.4 ± 113.7 Pa in unruptured aneurysms).

## Discussion

To the best of our knowledge, the present study provides the first comprehensive meta-analysis of WSS in ruptured IA. Our study shows for the first time statistical WSS maps of ruptured and unruptured IA based on the current literature. Even though there appears to be general consensus on aneurysm initiation, aneurysm rupture risk poses more vexing questions^28^. Our findings suggest that low WSS may be an important contributing factor to cerebral aneurysm rupture.

WSS differed between location in the aneurysm and the parent artery. PcomA aneurysms had lower average WSS (0.51 Pa), which was lower than that of MCA aneurysms (1.1 Pa). Multiple comparisons showed that basilar artery aneurysms had the lowest average WSS, being lower than that of anterior communicating artery (AcomA) aneurysms. WSS in AcomA aneurysms was lower than that in MCA aneurysms^29^.

Aneurysm wall degeneration increases from the neck to the dome, and pathological studies have revealed that ruptures commonly occur at the top of the IA. It has also been found that WSS is lowest at the apex of the IA rather than its neck^30^. Narrow-necked aneurysms are more likely to produce a recirculating and sluggish flow near the fundus with associated low and oscillating WSS vectors, which can drastically affect shear rates and accelerate wall degeneration^6,31^. This critically low-flow condition depends on aspect ratios and dome diameter, especially when the aspect ratio is >1.6^32,33^. In symmetric bifurcation, an inline inflow angle of 180° would also result in an area of stasis in the dome, leading to low WSS^34^.

Reduced velocities and flow recirculation are responsible for thrombus formation and further expansion of the aneurysm dome^35,36^. Ruptured aneurysms also have greater aneurysm areas under low WSS than unruptured aneurysms^7^.

It has recently been demonstrated that high WSS initiates aneurysm formation, whereas low WSS leads to spatial disorganization of endothelial cells and activation of the atherogenic and proinflammatory signal pathways^37^. The walls of ruptured aneurysms are fragile, possibly because of macrophage infiltration and the consequent apoptosis of smooth muscle cells and degradation of matrix proteins^38^. Nitric oxide (NO) is a key mediator of the effects of low and oscillatory WSS^39^. Moreover, selectin-mediated leukocyte rolling occurs at WSS near 0.4 Pa, and CFD analysis has repeatedly found that the WSS at aneurysm tips is below this value. Meanwhile, low WSS–induced expression of adhesion molecules, including vascular cell adhesion molecule-1 and intercellular cell adhesion molecule-1, reduced the rolling speed of inflammatory cells over the endothelium. The inflammatory cell–mediated degradation becomes even more pronounced upon the formation of intra-aneurysmal thrombosis, and favors aneurysmal rupture^40^. Atherosclerotic lesions also predominantly localize at sites that experience low shear, which can degrade the integrity of the aneurysm wall^41^.

Other studies have consistently reported that low WSS in the aneurysm region might help predict rupture^6,7,42^. Takao *et al*. found that the minimum WSS in the ruptured aneurysms group was half (absolute value 0.2 dynes/cm^2^) of that of the unruptured group^43^. Moreover, the flow characteristics just prior to rupture have been reported^44,45^, where both studies demonstrated low WSS on the aneurysm sacs before their rupture. Although the literature on this is scarce, some studies support the notion that WSS decreases sharply in the bleb region after blister formation^8,46^.

Some authors have reported that high OSI correlates with the etiology and location of atherosclerotic plaques, endothelial damage, aneurysm formation, and rupture. High OSI modulates the gene expression of endothelial cells to upregulate endothelial surface adhesion molecules, cause dysfunction of flow-induced NO, increase endothelial permeability, and thereby promote the rupture process^7,8,47^. In our analysis, six of fifteen reports found a positive correlation between high OSI and IA rupture. However, the pooled SMD between ruptured and unruptured groups showed high OSI in the ruptured group, but without significance. Nonetheless, some CFD studies have also suggested a role for high WSS in aneurysm rupture^48^. Therefore, discerning the actual cause predisposing these lesions to rupture is challenging, but it is likely the multiplicative effect of a handful of factors.

The role of WSS in rupture has not been conclusively elucidated. Endothelial cell responses to low WSS have been investigated in various *in vitro* studies, and excessively low focal hemodynamic WSS may be one of the main factors leads to decreasing resistibility and structural fragility of the aneurysmal wall^9^. A low shear magnitude can promote macrophage-related chronic inflammation and atherosclerotic changes. These atherosclerotic inflammatory changes and metalloproteinase production by macrophages can predispose wall to thinning and further rupture. As previous study found, atherosclerotic changes were seen more frequently in the wall of ruptured aneurysms^28^. Meanwhile, the activation of transcription factor NF-κB is prolonged under low WSS conditions. In addition, endothelial cells also upregulate the expression of genes involved in various processes such as cell growth and inflammation under low WSS^10^.

As for the specification of inlet flow conditions, different authors adopted different assumptions across different studies. Generally, it is very difficult to obtain the patient-specific boundary conditions in clinical applications. Those reports did not use patient-specific measurements when creating CFD models may have affected their results. Previous report also showed that the use of idealized assumptions would have larger and instable WSS results^11^. Normalization by parent vessel WSS generated from the same CFD simulation minimizes the dependence on inlet conditions, especially when patient-specific inlet flow conditions are unavailable. The findings of this study emphasize that the choice of generalized or patient-specific inflow boundary conditions results in variations in WSS magnitude.

Parent vessel reconstruction with flow diverter is becoming the preferred endovascular modality for giant and complex IAs as it is an effective supplementary to coil embolism for types of complicated aneurysms. Flow diverter attempts to redirect blood flow and reduces inflow and outflow of an aneurysm leading to aneurysm thrombosis and obliteration. Platelets also activate as they pass over the device and they strut into the aneurysm with a long residence time, thereby promoting thrombus formation. Flow diverters are expected to provide a scaffold which would promote the development of endothelial and neointimal tissue across the aneurysm neck while preserving patency of perforators and side branches. Unfortunately, delayed aneurysm rupture after flow diverter implantation has been reported without an understood mechanism which has tempered the enthusiasm for their widespread use. Xiang *et al*.^12^ suggest that flow diverter can generate stagnant aneurysmal flow and excessively low WSS, which may promote wall degradation via the inflammatory pathway. However, another study^13^ demonstrated that low post-implantation flow velocity, inflow rate, and shear rate are associated with fast occlusion times. Perhaps This reflects the race between formation of complete and stable thrombus and WSS-mediated inflammatory degradation of the aneurysmal wall. Meanwhile, the complication rate for unruptured aneurysms was significantly lower than that for ruptured IAs. Thus, the use of flow diverters in ruptured aneurysms poses a major clinical challenge. Larger case series are needed to define the safety role and indication of flow diverter application in such kind of clinical situations.

### Limitations

There were limitations to this study. First, publication bias are limitations that affect all meta-analyses. Indicating by I^2^ values, the substantial heterogeneity existed. Second, disparity of acquisition technique and inflow boundary conditions might also be responsible for discrepant WSS results in enrolled studies. Caution should be used in drawing conclusions from these comparisons.

## Conclusions

Decrease in local WSS may be an important predictive parameter responsible for IA rupture. The inconsistent findings of WSS value may be rationalized by small datasets, inconsistent parameter definitions, mechanistic complexity of IA rupture, and the compromises adopted in CFD simulations. Facilitating more accurate predictive models for IA rupture risk assessment from CFD in future studies will likely require better classification of aneurysms based on aneurysm location, morphology, perienvironment, and patient population, and the incorporation of cell biology, matrix biology, and aneurysmal wall imaging.
