# Author Correction: Association of wall shear stress with intracranial aneurysm rupture: systematic review and meta-analysis

**DOI:** 10.1038/s41598-018-23445-9

**Published:** 2018-03-22

**Authors:** Geng Zhou, Yueqi Zhu, Yanling Yin, Ming Su, Minghua Li

**Affiliations:** 1 Department of Diagnostic and Interventional Radiology, Shanghai Jiao Tong University Affiliated Sixth People’s Hospital, Shanghai, China; 2 Department of Anesthesiology, The Military General Hospital of Beijing PLA, Beijing, China; 3 Shandong Academy of Chinese Medicine, 7 West Yan Zi Shan Road, Lixia, Jinan China

Correction to: *Scientific Reports* 10.1038/s41598-017-05886-w, published online 13 July 2017

The original version of this Article contained a typographical error in the corresponding author’s email address: ‘liminghuayx@126.com’ should read ‘liminghua12@163.com’.

This error has now been corrected in the HTML and PDF versions of the Article.

